# Hospital Meetings, &C.

**Published:** 1898-06-11

**Authors:** 


### HOSPITAL MEETINGS, &C.

### THE LONDON HOSPITAL.

#### The New Out-Patient Regulations.

The governors of The London Hospital came to a very important decision at their quarterly court (which was held on Wednesday, the 1st inst., at the hospital, under the chairmanship of Mr. J. H. Buxton) as to the regulations which shall in future govern the admission of out-patients, and also as to taking the first step towards the abolition of governors' letters. The meeting was a well attended one, and the proceedings were characterised by a wonderful unanimity considering the importance of the subjects for discussion.

After the adoption of the report of the house committee, which was moved by Mr. J. H. Buxton, seconded by Mr. J. H. Hale,

The Hon. Sydney Holland moved the following resolution:—

"That the generous offer of £25,000 from an anonymous donor be accepted with grateful thanks by the governors of the London Hospital, and that the conditions attached thereto, namely, (1) that, subject to the regulations which shall be made from time to time by the house committee, those out-patients who can afford to pay for them be no longer supplied with medicines and bandages free; and (2) that governors' letters be not issued to any future governors or subscribers, be accepted."

He said that he had to propose a great change in the management of the hospital, a proposal which the House Committee unanimously recommended the governors to adopt. The governors knew what a very great difficulty the out-patient department had always been at The London Hospital. Year after year the numbers had increased, until now the accommodation was utterly inadequate. Two matters had brought this question to a head recently. The first was the very serious position of the hospital financially. He was not exaggerating when he said that if the legacies to that hospital continued to fall off as they had been doing, the hospital could not continue under its present conditions—it would have to very largely reduce its work. It was true they had got as a result of their appeal about £5,000 a year in new income, but that was only exactly half what was needed. The House Committee had, therefore, unanimously come to the conclusion that the time had come when it was impossible for them to go on supplying medicines and bandages free to those out-patients who could afford to pay for them. It was said that if a man attended a hospital who could afford to pay a medical practitioner it was an abuse of charity. He would go farther, and say that it was an equal abuse for a man who attended a hospital not to pay for the medicines and bandages supplied to him if he could afford to do so. They were perfectly willing to build that huge building, they were perfectly willing to supply all medical and surgical skill, all the enormous cost of upkeep, all the nursing, and everything else connected with that great hospital free of cost, because it would be impossible for the patients to pay for these things themselves; but they were not any longer willing to supply patients who could afford to pay for them with their medicine and bandages in the out-patient department. The second reason which influenced the members of the House Committee to propose that change was that a generous man had offered a sum of £25,000 for rebuilding that department on two conditions—first, that all out-patients who could afford it should pay for their medicines and bandages; and secondly, that governor's letters should not be issued to any new governors. It was the duty of the governors, as practical men and as men who devoted a great deal of time solely for the benefit of the poor people of the East-end, to see that in carrying out these recommendations no hardship was done to the patients themselves. Therefore, the regulations governing these points which the committee had drawn up had been framed very carefully indeed. Briefly, the regulations are these: All persons attending the out-patient department would be charged a small sum—threepence—for their medicines and bandages; anyone who claimed exemption on the ground of poverty would be given medicine or bandages free; the members of the staff would be allowed to exempt any patient in cases where they thought the small payment for medicines and bandages would keep that patient away in the future; children were also to be free, for it was thought that the parents might not give them the money to buy their medicines with, and the committee did not wish the children to suffer in any way. It was equally the desire of the committee that this change should not be unfair to the medical practitioners in the neighbourhood. He for his part could not see how it could be more unfair to the medical practitioners for the hospital to charge something for medicines or bandages than it was for the hospital not to charge anything at all. On the other hand, the charge of 3d. would probably result in a lessening of the numbers attending the out-patient department, and patients therefore would go to the medical practitioners. Therefore, if the change had any effect on the medical practitioners it would be to benefit them. Then any patient coming from an outside medical practitioner or from a provident dispensary for a consultative opinion would be supplied with his medicines and bandages free. Many difficulties and objections had been raised, but they had all been matters of prophecy and not of experience. He would not deny that the staff of the hospital were against the change. He was very sorry for it, because the House Committee did not like differing with gentlemen to whom they were so much indebted, but he ventured to think that this was a matter for the House Committee, who were responsible for the finances of the hospital, more than for the staff, provided every possible care was taken that the patient should know that he was not paying his 3d. for his treatment, but solely and simply for the medicine and bandages he received. As to the question of letters, he did not think that there could be any doubt that the proper passport for admission to the hospital should be illness and injury. It was possible they might find some few people who would say, "Very well then, as I have no letters given to me; as I may recommend the whole world to come to The London Hospital instead of the six people I formerly could recommend; as my bit of patronage is taken away from me I won't subscribe at all." People who would make that the excuse for not subscribing would soon find another, and in either case they would not long continue as subscribers. Surely, therefore, it was better to do right and lose some of those people now than to continue the present system and lose them eventually.

Sir Edmund Hay Currie, in seconding, was sure that if the patients themselves were asked their opinion they would prefer to pay something for their medicine and bandages, and so feel that they were helping their own hospital.

Mr. Loftus, who represented the East End Tradesmen's Association, established to assist its members to become life governors of The London Hospital, was afraid that the abolition of letters would end in the closing of the association, as one of the inducements to become members of that association was the fact that the life governors had the privilege of recommending patients.

Mr. Parker, who said he was a large collector in the East End of London for an institution for distributing letters to hospitals, agreed with Mr. Loftus. He suggested that life governors should receive letters whilst annual subscribers did not.

The Hon. Sydney Holland said that the representatives of the working men's associations under the new regulations as to letters still became governors of the hospital, and so retained a hand in the management of it. He was sure that the subscriptions to the working men's associations were not given merely with a view to what they could get back for them. He thought subscriptions to hospitals should be put on a higher line than the question of buying and selling. As a matter of fact, at the Poplar Hospital, where a similar course to the one proposed had been adopted a short time back, the working men's associations had continued to be subscribers, larger subscribers if anything than before. That was also the experience at St. Thomas', Guy's, and St. George's.

Mr. Loftus said that whatever course was taken he would continue to do his best for the hospital.

The resolution was carried unanimously, and

The meeting shortly afterwards terminated.

### JEWS' HOSPITAL AND ORPHAN ASYLUM, WEST NORWOOD.

Five years ago a Children's Orphan Aid Society was started in connection with the Jews' Hospital and Orphan Asylum. This society had for its object to enlist the practical help in favour of the asylum of children and young people under the age of 21, who, by payment of one penny a week—a sum within the means of every comfortably circumstanced child—constituted themselves members of the Children's Orphan Aid Society. Several of the religious centres of the Jewish community in London formed branches of this society from the young members of their congregations, who each promised to pay the subscription of one penny per week; and, so soon as each of these branches had a sufficient number of members to represent a subscription of £10 10s., they were entitled to elect one of their members to serve as a life governor of the Jews' Hospital and Orphan Asylum. Some of the branches have sent in as many as five and six life governors each every year, representing an income of between £52 and £63 annually to the institution, because once the children have joined the Orphan Aid Society it is a point of honour with them to continue to belong, and new subscribers come in from time to time, being induced thereto by the example of other children with whom they may be thrown in contact who may be members of this society. The work of treasurer and secretary of each branch is done voluntarily by members of the community from which the particular branch is formed, the minister of religion in most cases being the chairman, and the cost of collection of the subscriptions is reduced to a minimum by some of the child members undertaking to be responsible for the collection of subscriptions from their friends.

On Sunday, June 5th, all the members of the Children's Orphan Aid Society were invited down to the Jews' Orphan Asylum at West Norwood. Special trains were run from Victoria, and the whole institution was *en fête*. A very enjoyable time was spent by the little visitors, who were entertained in various ways by the inmates of the asylum. There was a pretty display of drill both by the boys and girls, and the boys' band was also much appreciated, its performance being indeed very creditable, seeing that the boy musicians are all under thirteen years of age, this being the age at which the boys leave the asylum to become apprenticed to some trade. The girls remain at the institution either till they are fifteen or sixteen, according to whether they intend to train for domestic service, in which they are instructed at the asylum, or for some other branch of work. There are about 300 children at present in the asylum, 125 girls and the remainder boys. There are carpenters' workshops for the boys, cookery and dressmaking classes for the girls, and there is also a large laundry on the premises where the washing of the whole establishment is done by the laundresses, who also instruct the older girls in laundry work. There is also a fine library of good standard works, and a good supply of books for the younger children. All the children look well and sturdy, and there was only one child in the infirmary ward, the institution being in practice remarkably healthy. The little visitors were conducted in parties over the institution by some of the inmates, who explained everything with much enjoyment, to the great interest of their guests, who were also allowed to see all the inmates of the institution at their tea, during which there was an organ recital by one of the masters. Altogether the Jews' Orphan Asylum should benefit considerably in the future by the interest which has been awakened in the minds of many of their young supporters, as well as those of older visitors, who were attracted by curiosity to see the institution on this occasion, and who, it is hoped, may often go there in the future from the desire to help in a practical manner the orphan children.

